# Dental Care and Education Facing Highly Transmissible SARS-CoV-2 Variants: Prospective Biosafety Setting: Prospective, Single-Arm, Single-Center Study

**DOI:** 10.3390/ijerph19137693

**Published:** 2022-06-23

**Authors:** Andrej Thurzo, Wanda Urbanová, Iveta Waczulíková, Veronika Kurilová, Bela Mriňáková, Helena Kosnáčová, Branislav Gális, Ivan Varga, Marek Matajs, Bohuslav Novák

**Affiliations:** 1 Department of Stomatology and Maxillofacial Surgery, Faculty of Medicine, Comenius University in Bratislava, 81250 Bratislava, Slovakia; marek.matajs@fmed.uniba.sk; 2 Department of Orthodontics and Cleft Anomalies, Dental Clinic 3rd Medical Faculty Charles University, Faculty Hospital Kralovske Vinohrady, 10034 Prague, Czech Republic; wanda.urbanova@gmail.com; 3 Department of Nuclear Physics and Biophysics, Faculty of Mathematics, Physics and Informatics, Comenius University, Mlynska dolina F1, 84248 Bratislava, Slovakia; iveta.waczulikova@fmph.uniba.sk; 4 Faculty of Electrical Engineering and Information Technology, Slovak University of Technology, Ilkovicova 3, 81219 Bratislava, Slovakia; veronika.hanuskova@gmail.com; 5 1st Department of Oncology, Medical Faculty, Comenius University, St. Elisabeth Cancer Institute, 81250 Bratislava, Slovakia; bela.mrinakova@ousa.sk; 6 Department of Simulation and Virtual Medical Education, Faculty of Medicine, Comenius University in Bratislava, Sasinkova 4, 81272 Bratislava, Slovakia; helena.svobodova@fmed.uniba.sk; 7 Department of Genetics, Cancer Research Institute, Biomedical Research Center, Slovak Academy Sciences, Dúbravská Cesta 9, 84505 Bratislava, Slovakia; 8 Department of Oral and Maxillofacial Surgery, Medical Faculty, Comenius University, University Hospital Bratislava, 81499 Bratislava, Slovakia; brano.galis@gmail.com; 9 Institute of Histology and Embryology, Faculty of Medicine, Comenius University in Bratislava, 81372 Bratislava, Slovakia; ivan.varga@fmed.uniba.sk

**Keywords:** dental education, biosafety, dentistry, orthodontics, sustainability, COVID-19, infection, prevention, teledentistry, UVC, virucidal oil dispersion

## Abstract

With the arrival of the highly transmissible Omicron variants (BA.4 and BA.5), dentistry faces another seasonal challenge to preserve the biosafety of dental care and education. With the aim of protecting patients, students, teachers and healthcare professionals, this paper introduces a prospective sustainable biosafety setting for everyday dental care and education. The setting developed by dental clinicians, epidemiologists, and teachers of dentistry consists of a combination of modern technologies focused on the air-borne part of the viral pathway. The introduced biosafety setting has been clinically evaluated after 18 months of application in the real clinical environment. The protocol has three fundamental pillars: (1) UVC air disinfection; (2) air saturation with certified virucidal essences with nebulizing diffusers; (3) complementary solutions including telehealth and 3D printing. A pseudonymous online smart form was used as the evaluation method. The protocol operates on the premise that everybody is a hypothetical asymptomatic carrier. The results of a clinical evaluation of 115 patient feedbacks imply that no virus transmission from patient to patient or from doctor to nurse was observed or reported using this protocol, and vice versa, although nine patients retrospectively admitted that the clinic visit is likely to be infectious. Despite these promising results, a larger clinical sample and exposition to the current mutated strains are needed for reliable conclusions about protocol virucidal efficiency in current dental environments.

## 1. Introduction

Clinical dentistry as well as dental education experienced more than two difficult years of the SARS-CoV-2 pandemic. Initially, some experts expressed doubts about the potential of this new virus to cause a global pandemic with significant socio-economic impacts. This has also affected the domain of the dental community, including clinical care and education [1,2,3,4,5,6,7,8].

The world is now confronted with highly transmissible variants of SARS-CoV-2 (Omicron strains BA.1 overtaken by more transmissible BA.2) causing COVID-19, although these are fortunately less virulent than previous strains. Strains BA.4 and BA.5 are at our doorstep suggesting a possible autumn breakout. The dentistry sector is now facing these highly transmissible SARS-CoV-2 variants with currently highly protected, albeit waning, immunity in the population after mass vaccinations. There is a demand for a biosafe workflow in dental care and education. Even though COVID-19 is currently less lethal than ever before, the overall risk of infection, post-viral syndrome (long COVID-19) remains higher than at the beginning of the pandemic. So far, dental professionals have been able to anticipate the risks of incoming waves and their seasonality. Biosafety measures in dental offices have been repeatedly revised to protect patients and healthcare workers [2,9,10,11,12,13,14,15,16]. Dentists have changed their behavior and adapted their workflows [1,2,17].

Dental care and education are characterized by close personal contacts and treatment procedures that produce aerosols. Dental healthcare professionals, including dentists, dental assistants, dental hygienists, and nurses were aware of the high risk of exposure in the early stages of the COVID-19 pandemic [18,19]. The fact is that dentists are at a high risk of contracting COVID-19 from their patients because of its transmission by respiratory droplets and the use of dental handpieces that can generate aerosols [20,21], as well as the physical proximity of their patients [19,22,23]. Understanding the significance of aerosol transmission and its implications in dentistry can facilitate the identification and possible correction of negligence in daily dental practice [24]. The mitigation of particles that can carry the virus, and thus the mitigation of the risk of pathogen transmission in dental offices, often confirm the high effectiveness of personal protective equipment in protecting patients and dentists from aerosols [25].

Dental education struggling with the biosafety of students, patients and educators was characteristic, along with its high adaptability to implementing modern technologies, including online educational platforms, teledentistry diagnostics and various e-learning tools [8,26,27,28]. In 2021, Varvara et al., published a study designed to determine the undergraduate student perception of e-learning educational methods. The student feedback showed significant appreciation (*p* < 0.05) of the new methods, although a lack of practical training was significantly perceived as an important problem in the structure of their new curriculum [29].

In March 2020, nearly 200,000 dentists in the United States closed their offices to patients in fear, fueled by concerns that aerosols generated during dental procedures are potential vehicles for transmission of respiratory pathogens through saliva [30]. The findings published by Meethil et al., in the Journal of Dental Research [31] suggest lower risks for transmission of the SARS-CoV-2 virus during dental procedures than anticipated.

Preprocedural rinsing is one of the biosafety precautions introduced later in this work. The authors have learned from their clinical experience that this can occasionally lead to a patient developing cough, and therefore providing the exact opposite of the intended effect. While preprocedural rinsing has been encouraged since the onset of the pandemic, the guidelines on which antiviral to use were unclear. The American Dental Association (ADA) initially recommended 1.5% hydrogen peroxide and 0.2% povidone iodine for use as an antiviral prerinse [32,33]. When actual antiviral testing of these commercial rinses on SARS-CoV-2 finally became available, a different picture began to emerge. While the ADA recommended 1.5% hydrogen peroxide as an antiviral prerinse, the Centers for Disease Control and Prevention (CDC) advised that 1.5% hydrogen peroxide needs 18 to 20 min to inactivate rhinovirus, the virus that causes the common cold [34]. In October 2020, an extensive review inspecting the antiviral efficacy of hydrogen peroxide mouthwash was published in the Journal of Hospital Infection [35]. The authors concluded that: “there is no scientific evidence supporting the indication of hydrogen peroxide mouthwash for control of the viral load regarding SARS-CoV-2 or any other viruses in saliva.” [35]. As a result of this knowledge and additional in vitro and in vivo tests, the Royal College of Dental Surgeons and the Canadian Dental Hygienists Association have advised all their constituents to discontinue the use of hydrogen peroxide as an antiviral prerinse [36]. In August 2020, the Antiviral Research Institute of Utah State University piloted a study of the antiviral efficacy of several oral rinses against SARS-CoV-2 [37]. Of all the rinses evaluated, 0.12% chlorhexidine gluconate and 1.5% hydrogen peroxide were inadequately effective, even after 60 s of exposure. While 0.2% povidone iodine performed slightly better, the only rinse that completely inactivated SARS-CoV-2 was a 100 ppm molecular iodine rinse. It was completely effective within 30 s. None of the iodine rinses were cytotoxic, but the hydrogen peroxide and chlorhexidine gluconate rinses were. At present, molecular iodine rinse is the clear evidence-based winner as a prerinse for SARS-CoV-2. This is particularly important considering that many other oral rinses are neutralized in the presence of saliva [38,39,40,41,42,43]. Oral rinses are a regular part of various dental biosafety protocols.

Dental care is often provided to oncological or other immunocompromised patients. It is crucial that precautionary measures are implemented so that these patients can be treated in a safe environment. A timely adaptation of clinical workflows and implementation of practice modification measures was observed throughout the world [15,44,45,46,47]. These, with the arrival of significantly more infectious Omicron-strains, need to be revised [45]. Moreover, dental care is essential in dealing with toxicity of anti-cancer treatments such as oral mucositis, xerostomia, trismus, osteoradionecrosis, and opportunistic infections [48]. Optimal safety protocols must be applied to minimize the risks in this population during dental care and education.

Current preventive biosafety measures often consider the possible specific intraoral manifestation of COVID-19. Triad xerostomia, taste and smell dysfunction, and oral mucosal lesions were identified as common manifestations with previous variants; however, with still controversial causality in omicron variants, xerostomia, taste, and smell dysfunctions are no longer common symptoms. A causal relationship between oral lesions and COVID-19 has been proven [49,50,51,52,53].

The pandemic has had a significant impact not only on dentists and their colleagues [54], but also on patients’ mental well-being. Frequently, the occurrence of depression, anxiety, stress, intrusion, avoidance, and hyperarousal were observed both in patients, as well as in healthcare workers [55,56,57,58,59].

In the beginning of the pandemic, the Hospital of Stomatology in Wuhan diagnosed nine dental staff members infected between January to February 2020 [19]. Chinese dental surgeons responded with set of recommendations for the biosafe management of dental care workflow in the context of the epidemic [22]. Since then, various recommendations and guidelines have been published on professional websites in several countries. Most dental healthcare professionals had a high level of awareness for general COVID-19 infection prevention and control guidelines [59]. For example, guidance was provided in the US (Centers for Disease Control and Prevention (CDC), American Dental Association), in Europe (European Centre for Disease Prevention and Control (ECDC)), in France (Health Ministry, French Dental Association) and in the UK (National Health Service, British Dental Association) [60]. One of the first renowned pandemic-dental events in European dentistry was the outbreak in North Italy in Lombardy. All of Lombardy’s dentists were evaluated with an online ad hoc questionnaire; 3599 questionnaires were analyzed. Of these, 502 (14.43%) participants had suffered one or more symptoms referable to COVID-19; 31 subjects were positive for the virus SARS-CoV-2 and 16 subjects developed the disease. Only a small number of dentists (*n* = 72, 2.00%) were confident of avoiding infection [61].

Several innovative biosafe approaches of dental diagnostics or treatment were introduced, for example telehealth solutions such as Dental Monitoring^®^ (DM) (Dental Monitoring Co., Paris, France) [6,62,63,64,65,66,67]. Revisions of the then-established protocols in dental healthcare were simple. Dental professionals, in their early efforts to adapt their biosafety measures, typically performed a web search conducted in the main databases of the scientific publications, focused mostly on oral rinsing and limitation of in-office aerosol production. This early research often led to possible revisions of biosafety and disinfection protocols in the dental offices [1,2]. Two years of ongoing pandemic has changed the practice of dentistry forever, and some of these changes have made dental care more time-consuming, difficult, and costly due to the possible pathways of transmission and mitigation steps needed to prevent the spread of the infection.

Despite the widespread anxiety and fear of the devastating health effects of earlier COVID-19 (2020–2021), only 61% of dentists have implemented a fundamental modification to their treatment protocols. Currently, facing the highly transmissible Omicron strains, as an urgent matter of public health, all dentists must identify the additional steps they can take to prevent the spread of air-borne infection [21].

The clinical practice biosafety guidelines, developed during the first year of the pandemic, offer recommendations which guide dental staff in providing safe dental care in the clinical environment. Such recommendations must be updated as new evidence of virus properties arises [68,69]. There is a high level of agreement between different dental specializations about the necessary preventive measures of the routes of transmission. Published data regarding the survivability of the virus on innate objects vary substantially [70]. Nevertheless, due to the wearing of personal protective equipment (respirators, gloves, masks, eye shields, and gowns) and use of disinfection procedures, this risk can be significantly mitigated. Research published by Estrich et al., found in June 2020 that during the first wave of pandemic only 0.3% of surveyed dentists had a probable COVID-19 diagnosis, of these 82.2% were asymptomatic. The most reported health problems among dentists during the pandemic were anxiety and depression [21,71].

The primary aim of most biosafety protocols is to prevent any cross-contamination while allowing the provision of urgent and emergency dental care. Aerosol-producing and other elective procedures should be avoided in the periods of outbreaks of unknown variants [17,72,73,74,75]. Various biosafety protocols detail the safety and operational measures to be taken, while providing dental care in the COVID-era. Falahchai et al. [76] published a comprehensive protocol regarding dental care during the COVID-19 outbreak. The point in the outbreaks caused by new, not well researched variants, is that these might bring dangerous long-term health hazards enabled by new mutations.

With the currently waning post-vaccination immunity worldwide, together with less cautious behavior of the general public, there is a high probability of infections providing more opportunities for further virus mutations. The Omicron variant BA.2 will only be substituted with more transmissible strain. Hopes for guaranteed declining virulence of future mutated strains are “wishful thinking” rather than an evidence-based theory. As evidence mounts that the Omicron variant is less lethal than prior strains, one of the frequently cited explanations is that viruses always evolve over time to become less virulent. This theory has already been soundly debunked. During surges of unexplored mutated variants, dental treatment might be limited to patients with urgent or emergency situations. Patients should be provided with separate waiting and operating rooms to minimize the risk of transmission of infection and treatment should be provided with the same protective measures regarding Personal Protective Equipment (PPE) for the dental clinicians and staff [76].

The last two years of pandemic in dental care showed changes in attitude from initial negligence of possible serious impacts of the virus spread, up to panic precautions. After a year, the variables of the pandemic situation have changed with the implementation of mass vaccination. This represented an important milestone [77,78], suggesting a possible end-game scenario for the COVID-19 pandemic. These expectations were facing disappointment with the arrival of the Omicron variant. The imagined race between SARS-CoV-2 mutations and vaccine rollouts was slowed down with negative perception of people afraid of possible vaccination side effects. A recent study from the Czech Republic proved the distribution of side effects among Czech healthcare workers as highly consistent with the manufacturer’s data. The overall prevalence of local and systemic side effects was higher than the manufacturer’s report [78]. Current data suggest that Omicron capabilities to evade natural human immunity, protective effects of distributed vaccines [79,80] and its resistance to current monoclonal medication [81,82] make the Omicron variant a true dilemma. According to a recent study by Dr Michael Chan Chi-wai, from December 2021, it can infect faster and better than Delta in human bronchus, but with a less severe infection in the lung [83]. Due to its newly gained strong ability to infect ACE2 in mice, it is predicted that this variant is here to stay until pushed out with a more transmissible strain [84].

A recent study of the American Dental Association published by Araujo et al. [23] suggests that US dentists show a high level of adherence to enhanced infection control measures in response to the ongoing pandemic, resulting in low rates of cumulative prevalence of COVID-19. With the spread of “Omicron-like” mutant strains, the likelihood of recurrent infection raises some doubts about whether vaccination alone will provide long-term immunity against COVID-19 and its future variants. Furthermore, several mutations in the receptor binding domain and S2 are predicted to impact transmissibility and affinity for ACE-2 that might be relevant for the adaptation of biosafety protocols [80].

From this perspective, the aim to provide biosafe dental workflow in orthodontic or any other dental practices must consider the higher transmissibility of the virus and higher number of asymptomatic carriers. The expectation that when the virus spreads naturally it will diminish or become less virulent is not possible, nor is it certain that further mutations will be harmless. Therefore, the effective biosafety protocol must be sustainable. Based on these facts, various simulations and modelling data, dental practices may never return to “normal”, former routine operation even after global vaccination would be somehow successful as there would still be a significant risk of outbreaks of infection with new mutant strains. Variable, multi-level measures will still be required, depending on the local COVID-19 cases rate, to secure safe dental care provision [45].

Biosafety protocols, applied a year after the pandemic outbreak, are not significantly different from the ones effective today, although the virus transmissibility and symptoms have changed significantly. This work introduces a prospective setting of a combination of field-tested technologies for enhanced clinical biosafety of orthodontic workflow in confrontation with surging cases of Omicron variant infections [79,83,84,85]. The effectivity and sustainability of this protocol lies in its simplicity and the fact that despite new variants being more invasive to human immunity, their air-borne pathway is equally obstructed with masks, and indoor biosafety measures.

Omicron SARS-CoV-2 (B.1.1.529) and its sublineage BA.2 remain variants of concern [69,86] and they define a new chapter in the COVID-19 pandemic. This highly mutated strain has more than 30 mutations, several of which overlap with those in the Alpha, Beta, Gamma, or Delta variants of concern. These mutations and deletions are known to lead to increased transmissibility, higher viral binding affinity, and higher antibody escape. The long-term potential of the new variants is not certain yet [79,80,81,84,85,87,88,89,90] and preliminary research publications suggest it is from 2 to 4.2 times more infectious than the Delta variant [85]. BA.2 is more transmissible, more replicative in human nasal epithelial cells and more fusogenic than BA.1 [86]. It evades not only human natural immunity but also immunity from vaccines [84], and a number of laboratory studies have previously shown a reduced ability of approved vaccines to neutralize the new variant.

The ability of this variant to evade immunity from previous infections in contrast to the Delta and Alpha variants was presented in population-level evidence [90], and thus, this suggestion is epidemiologically important in countries with huge amounts of previously infected cases, such as Slovakia. Preliminary evidence [87,91,92] shows that there is an increased risk of reinfection of the Omicron variant and this is probably caused by Omicron escaping neutralization antibodies [93]. Even after two doses of mRNA vaccines, higher transmissibility of Omicron was also observed in Norway [94]. The booster dose may not prevent people from becoming infected, but the observed symptoms were mild or moderate [95,96]. The time since the second or booster vaccination plays a role in effectiveness against symptomatic disease, as noticed in United Kingdom [97].

Based on these evaluations of Omicron strains, each new VOC results in uncertainty; there is a struggle to reduce transmission, discuss vaccines efficacy, supply elective-care and prevent long COVID-19 complications. Thus, it is not only vaccines that play a key role in preventing COVID-19 spread, but also solutions such as biosafety protocols are complementary and highly demanded.

The risk of transmission of pathogens in the dental office resulting in an infectious disease is still unknown; it seems to be limited in developed countries, but it cannot be considered negligible [98]. Current biosafety settings are organized into five distinct areas of pandemic control, comprising:

(1) Planning and protocols;
(2) Patient screening;
(3) Preparation of facilities;
(4) PPE and infection control;
(5) Aerosol control.

Research published by Estrich et al. [71] showed that dental professionals have enhanced their infection control practices in response to COVID-19 and have benefited from a greater availability of personal protective equipment. Most practicing dentists (72.8%) used personal protective equipment according to interim guidance from the Centers for Disease Control and Prevention.

As the pandemic situation will develop towards more infectious variants, new considerations, new protocols, and new mechanisms will be implemented in the dentistry profession, including the teledentistry approach [99].

The possible lower lethality of the Omicron variant in combination with the significantly higher ability to infect and create asymptomatic carriers might be dangerous. The effects of COVID-19 are very diverse and they vary from individual to individual. Many patients develop long-term disabilities, such as pulmonary, cardiovascular, hematological, renal, gastrointestinal, reproductive, psychological, and central nervous system problems, which can last from months to years [100]. The blood–brain barrier pathway can let the virus into the neural system and cause neuronal damage as well as neurodegeneration or long-term neurological and psychosocial consequences [101,102,103,104]. Cardiovascular problems (e.g., myocarditis, arrhythmias, myocardial damage) are also quite common [105] and recent studies have shown that SARS-CoV-2 infection also affects the human reproductive system, and especially the male reproductive system via the ACE2 receptor [106,107].

This work introduces a novel combination of biosafety measures for dental workflow adaptation to preserve biosafety in dental care confronted with highly transmissible air-borne viruses. The combination of the described procedures and technologies, especially setting with UVC and virucidal air-dispersed oils, can suppress the air-borne translation of the virus and has not been investigated yet. This biosafety protocol could provide a simple and long-term sustainable model for a biosafe dental workflow, as it can manage renewed and increased risks brought by the more infectious Omicron variant carried with asymptomatic infectious patients. The present study aims to determine its clinical reliability with retrospective identification of events where a dental procedure was performed unknowingly on an infected patient, the frequency of this occurring, and cross infection incidents.

The main goal of this patient-centered study was to evaluate introduced biosafety measures for orthodontic workflow as a prospective setting for prevention of in-office infections with air-borne SARS-CoV-2 variants. The secondary goal was to provide preliminary data for a larger study.

## 2. Materials and Methods

### 2.1. Main Objective and Study Design

The study objectives were to answer whether, under this prospective setting, infected patients were treated and if any infection with SARS-CoV-2 occurred in the monitored clinical environment.

This was a single-arm, single-center small-scale study set up in a particular clinic. Data were collected after 18 months of protocol in place with a pseudonymous online form. Everyone treated in the clinic within this period experienced the same conditions.

The primary planned outcome of the study was a confirmation of exposure of the environment and personnel to infected patients with no cross infections.

Due to known limitations of single-arm studies, the conclusions of this study can be limited. On the other hand, this design was the only viable choice for investigating biosafety measures, as the non-application of any preventive measures in pandemic would not be ethical. Alternatives for external control groups for this single-arm trial were considered; however, there is no comparative data available about how frequently patients become infected during orthodontic appointments.

### 2.2. Participants and the Environment

The protocol was applied and evaluated in the clinical environment of dental clinic with 4 doctors, 4 dental nurses and one manager.

During an 18-month evaluation period, approximately 2500 appointments were scheduled in a digital calendar for 160 different patients, not including their accompanying persons. Approximately 2230 appointments took place (others were cancelled or postponed mostly due to pandemic health precautions or travel complications).

The environment for dental care consists of fully separable rooms with independent air processing and separate Ultraviolet—C light (UVC) air sterilization systems. Each of the three rooms have an independent dental unit with a separate nurse position.

### 2.3. Brief Description of the Protocol

The key attributes of this biosafety protocol are:

- Efficient;
- Sustainable;
- Simple;
- Applicable in other dental specialties (despite orthodontic customization).

The fundamental backbone of this protocol is a combination of air treatment and new technologies. Air treatment is performed with a combination of hooded UVC sterilization and air disinfection with the creation of a virucidal air puffer made by a certified virucidal oil nebulizer/diffuser. Key technologies implemented as part of this protocol in the workflow are artificial intelligence in patient diagnostics, continuous and post-treatment monitoring, smart mobile patient coaching, 3D printing for aerosol control and some other techs.

The set-up of this protocol might be considered as a biosafety overkill; however, the current risks of airborne infection that could come from an asymptomatic carrier and circumvent immune defenses, causing a permanent health damage to someone else, is not negligible. With future, more transmissible, variants to come, professional dedication to preserving the highest biosafety level in the dental office and efficient air processing protocols will be needed.

This protocol is intended as a possible complement to existing methodologies. It represents more than a year of our interdisciplinary efforts dedicated to clinical testing and the implementation of various new technologies and working procedures to maintain the biosafety of dental healthcare. Dental care must be provided taking into consideration the patient safety as well as the safety of healthcare professionals.

This protocol was created as part of an interdisciplinary cooperation of epidemiologists, infectologists, dental surgeons, general dentists, orthodontists, and other healthcare professionals. It provides inspiration for wider clinical implementation and assessment. It is our contribution to solving this historical situation affecting safety and availability of oral care.

The fundamental principle of this protocol is full room volume air treatment with a combination of UVC and virucidal oil diffusion. Sterilization of the entire volume of air (for each patient) of the given treatment unit (room) by a combination of a UVC light and a created permanent virucidal buffer of certified disinfectant oils and special active FFP3 protective aids. The key additions to the protocol are fundamental changes in working practices in the provision of artificial intelligence and telemedicine. There are two main parts of the permanent daily protocol:

- Full room-volume air UVC sterilization for each patient ∗ total air volume per dental unit (separated room);
- Permanent air dispersed super small droplets of virucide oil—a protective puffer in recommended concentrations, created with disinfectant fogging machine (nebulizer).

Complementary technologies for digital workflow:

- AI video-scan evaluation (common checkups rendered obsolete)—Dental Monitoring;
- custom made IOS/Android app coaching the proper habits—StrojCHECK [12];
- 3D printed aerosol vacuum pump ending, that supports aerosol dispersion during dental procedures and other customized 3D printing allowing more.

### 2.4. Comprehensive Biosafety Protocol Description

#### 2.4.1. Introduction and the Focus of the Protocol

The aim of this protocol was to define safe and, at the same time, physically and economically sustainable measures for a longer period of the pandemic with the minimization of the impact on the provided healthcare in dental practice. The objective was to maintain a high quality of care, without a significant increase in the costs and with no tolerance to any exposure of the staff or patients.

Work on this protocol began on 6 February 2020, and it took us more than 9 months to experiment, test, and implement various new practices, technologies, and modifications to existing practices. Through final consultations with experts, it has taken on its present form. This protocol can be currently considered as useful and inspiring for clinicians looking for cost-effective solutions that can be sustainable in the long run. Despite all our staff being vaccinated, with the Omicron variant, they might be infectious and simultaneously not show clinical symptoms [79,80,81,82,83,85,87,88,89,90].

New and improved procedures have been implemented to develop this protocol in cooperation with renowned infectologists, epidemiologists and other experts. Examples of some implemented technological innovations are:

1. Online dynamic anamnestic forms (effectively replacing part of 4D clinical examinations);
2. Teledentistry Teleorthodontics—Dental Monitoring^®^ (DM) (Dental Monitoring Co., Paris, France)/https://dental-monitoring.com/ (accessed on 1 December 2021) and (StrojCHECK^®^, Bratislava, Slovakia, 3Dent Medical, www.osim.sk (accessed on 1 December 2021) [12];
3. Artificial intelligence in telediagnostics—active screening of patients in tandem with the doctor;
4. Special medical devices—active FFP3 shield respirator, BioVYZR, Toronto USA-based Vyzr Technologies, a shield that covers the wearer’s face and protects against droplets and pathogens. Powered Air Purifying Respirator (PAPR), a device typically used only in industrial and healthcare settings;
5. 3D printing of sterilizable devices (aerosol aspirators for surgical aspirators or individualized handles);
6. Two-phase air sterilization (diffusers of biocidal oils with UVC, NewAroma.sk).

#### 2.4.2. The Protocol Principles

This protocol does not inevitably change the procedures already established so far for sterilization of dental instruments, disinfection of surfaces and other routinely applied rules of each dental clinic. These remain, as the current pandemic is just one of the many infections we fight in asepsis in the dental clinic environment. Its key set of differences from HIV, EBV, HSV and other common viruses is the period of possible asymptomatic air-borne transmissibility. The advantage of modern orthodontic therapy with clear aligners is the relatively rare occurrence of the need for urgent treatment. In the case of urgent orthodontic treatment of a confirmed infectious COVID-19 patient, a bio-hazard protocol is applied as in HIV patients, in COVID-19 with an emphasis on the prevention of airborne spread. Regardless of whether the treatment involves aerosol dispersion (e.g., removal of attachments or fixed retainer or orthodontic auxiliaries such as distalizer, power-arms [108,109] or power caps), the treatment procedure differs from the standard protocol only in the fact that the patient has the last appointment of the day shift—as the last procedure on a given day.

Oral rinsing is not routinely recommended in our protocol for all patients. Only in the patients where intra-oral procedure is planned, iodine prerinse for 30 s is indicated. Rinsing does not address the nasopharyngeal region with the largest “virus load” (reported in the nasopharynx). From our practical experience, the recommended 30 s rinsing often led to a “run-in” and subsequent coughing of the patient and thus unnecessary contamination of the space. This protocol recommends careful rinsing only while attachments are being replaced or cleaned, during bio3dir removal and “powerCaps”, “powerArms” removal, interproximal recontouring or dental hygiene procedures. Prior to these procedures, the patient rinses the mouth for 30 s.

This protocol works on the premise that either patient, nurse or doctor are infectious. This is the reason the protocol includes:

1. Minimization of time exposure (shortened duration of treatment, replacement of unnecessary physical visits of the patient via AV technologies, ordering for the exact time with preparation of everything necessary for the procedure in advance).
2. Mechanical and physic-chemical prevention in the dispersion of the virus and its carriers into the space resp. decontamination (PPE, continuous sterilization of the air and virucide “puffer” in the air) et al.
3. Preventive (patient input filter—symptoms, smart-App tracking, continuous testing of healthcare professionals).

A slightly elevated temperature that is acceptable under this protocol is 37.8 °C (originally taken from CDC). The uniforms of the medical staff are rotated daily. They include all surface clothing including shoes and socks. No hazmat suits are used.

#### 2.4.3. Brief Description of Technologies Utilized in the Protocol

**Figure 1:**
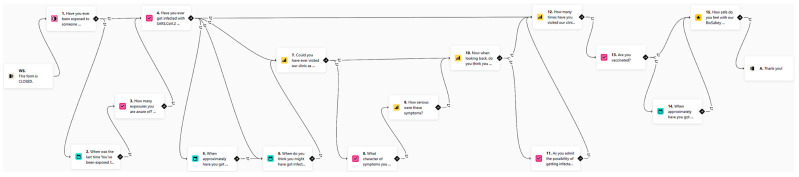
Example of logic behind the online smart form. English version of the survey evaluation in this protocol. URL: https://sangreazul.typeform.com/BioSafety-ENG (accessed on 15 December 2021).

**Figure 2:**
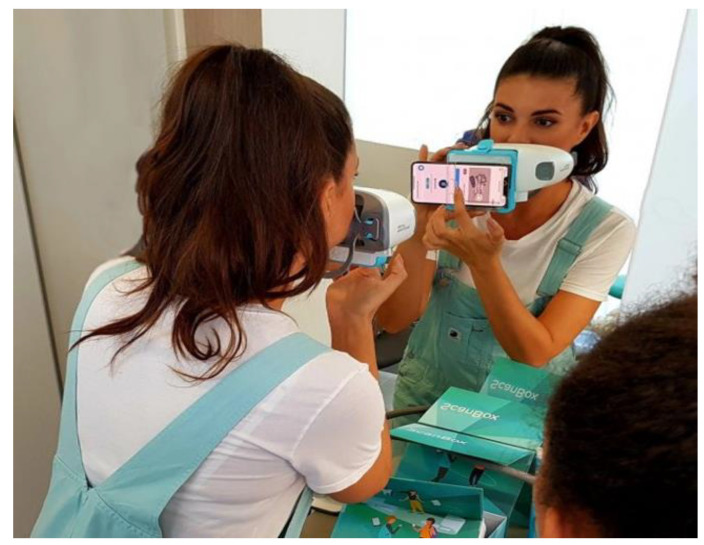
First Dental Monitoring session, in which the patient is educated with the nurse about how to use the free app in his/her own mobile in combination with the “scanbox” holder. Published with written consent of the person.

**Figure 3:**
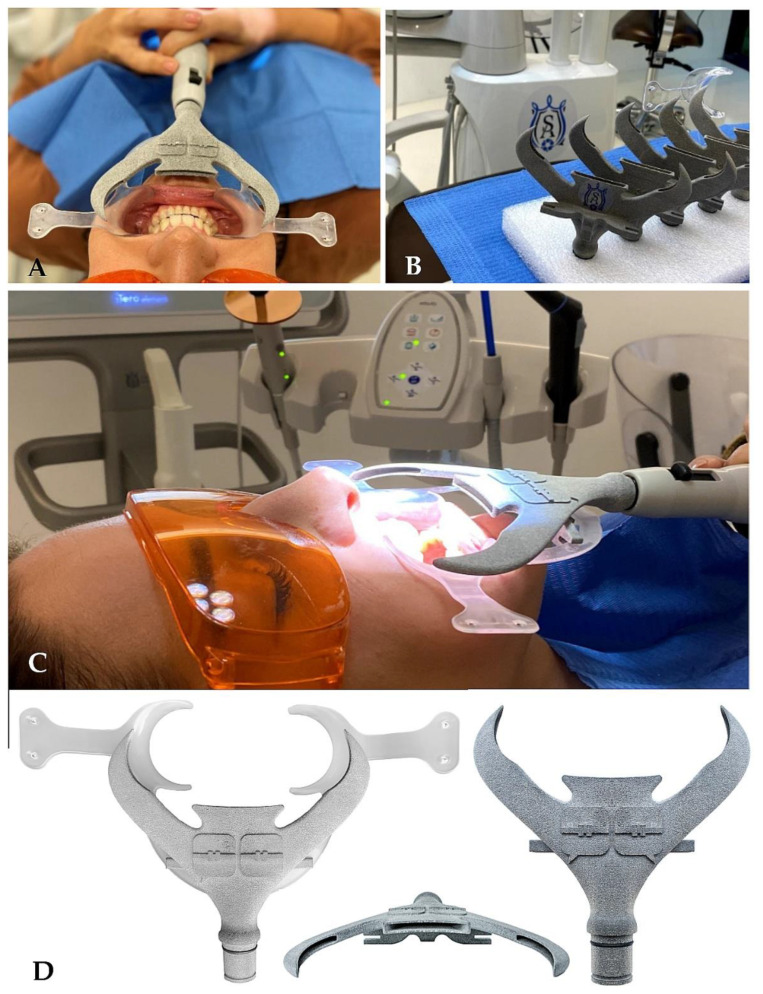
3D printed aerosol interception device. (**A**) Frontal view of 3D printed aerosol suction attached to surgical suction system and oral retractor. (**B**) With 3D printing, each office can be supported with adequate quantities. (**C**) Lateral view of “SUR-FACE” attached to conventional retractor. (**D**) View of “SUR-FACE” with and without cheek retractor and view of three suction openings.

The protocol implements the following technologies and practices:

1. Online dynamic anamnestic forms;
2. Artificial intelligence in dental monitoring;
3. Robotization of automatic surface disinfection;
4. A.I. smart patient app for treatment coaching;
5. 3D printing of sterilizable devices;
6. Regular COVID-19 testing of personnel;
7. UVC and diffused virucidal oil air treatment.

1. The use of online dynamic anamnestic smart forms such as Typeform (www.typeform.com, accessed on 18 May 2022) brings interactivity and order to communication with patients at home. The idea of online dynamic anamnestic forms is to use the patient’s own mobile phone not only for video communication (WhatsApp, Facetime), but also for outsourcing part of the examination. For example, with the proper instructions, a short selfie/video sequence of a natural smile can be captured by the patient and provided within an online smart form. The anamnesis itself is very important in reducing the risk of transmission, the proportion of asymptomatic patients with COVID-19 was less than 30% in the number of children with the highest proportion of asymptomatic infection. The identification of symptomatic patients is the first and relatively most effective method of risk reduction. Since the anamnesis is taken remotely, neither staff nor co-patients are exposed to the risk of infection, nor is there any contamination of the surfaces in the waiting room. The use of dynamic forms from Typeform is a suitable choice for this protocol. Remote screening of COVID-19 symptoms by application has been identified as a suitable method for detecting COVID-19 infection. Even the survey form for this biosafety protocol clinical evaluation was created as a smart form. It asks a set of questions that differs according to the answers given. The logic behind it is shown in Figure 1.

2. Artificial intelligence (AI) technology implemented in the form of the Dental Monitoring software uses the patient’s mobile phone for regular “scanning”, either to assess the course of treatment or to screen a growing patient by monitoring their development or monitoring retention stability after cessation of treatment. A special holder allows the patient to record a video of their own teeth. Paradoxically, it allows more regular and even more thorough inspection, as using artificial intelligence allowed us to summon the patient to an appointment only when necessary (Figure 2). Each video scan first evaluates using AI and then it alerts the doctor only to the monitored situations. The form of our workflow has thus changed fundamentally, and healthcare professionals spend a large part of the day reviewing the outputs of artificial intelligence, which, in turn, extremely efficiently evaluates huge volumes of data, such as video scans of our patients’ oral cavities. It is not humanly possible to evaluate the hundreds of video scans that patients regularly make. The “brute force” of this technology is ideal for identifying situations requiring human intervention.

3. Robotized around-the-clock surface disinfection technology is also used. It should respect all existing guidelines with an emphasis on consistency and differentiation of surfaces and a higher frequency of cleaning (after each patient). After entering, the patient should only be in contact with the necessary surfaces, and disinfect her/his hands first. Frequent cleaning of surfaces in the clinic, after each procedure, should include frequently inspected surfaces such as door handles, keyboards, and mice. There is also the addition of floor cleaning by robotic vacuuming with a disinfection mop, in our case, the iRobot Braava jet m6, which is suitable for up to 100 m^2^. The CDC recommends applying standard virucidal disinfectants to potentially contaminated surfaces to prevent the spread of SARS-CoV-2 infection. The work on the susceptibility of human coronaviruses and SARS-CoV is expected to be highly effective in SARS-CoV-2, which is relatively resistant to environmental conditions and remains infectious on smooth surfaces such as metal and plastic for many days.
4. Self-developed smart mobile application technology is used for patient treatment coaching and remote discipline support. The authors of this paper have been gradually developing iterations of the free telehealth smart patient application for patients in orthodontic treatment with clear aligners, to support proper habits/stereotypes. This app allows for better remote management of the patient and possible reduction in the frequency of visits to the dental office. The application educates and motivates the patient to behave responsibly [12]. For example, the app provides motivation and coaching for more frequent cleaning of teeth and aligners and in general it improved patient compliance.
5. 3D printing technology supports the presented biosafety protocol with specific sterilizable aerosol aspirators (Figure 3A–D). These devices are printed by a MultiJet Fusion 3D Pro Printer and are sterilizable at 121 °C in autoclave. This aerosol interception device is called “SUR-FACE” and was developed by an Italian orthodontist because of the COVID-19 pandemic. It connects to a conventional 16 mm suction device. The material is polyamide and is compatible with any retractor with thickness ≤ 2 mm. The material is recyclable and supplied with five additional rubber seals. The one-time use of suction cup attachments minimizes the risk of transmitting infection with this tool. The sterilizability of these handpieces is critical. From clinical experience, it is very effective at containing aerosols produced during clinical procedures.

6. Regular antigen testing from saliva or other convenient form of testing for possible infectiousness of everybody in the team shall be employed because, with the arrival of Omicron-like strains, it is even more likely that a vaccinated healthcare professional would become a “supercarrier”.
7. Air-processing technologies are key to the presented biosafety protocol with two-phase air treatment: room air is the most likely vector and therefore a key element in the stopping of transmission of infection. The transmission of SARS-CoV-2 infection occurs mainly through droplets that fall relatively rapidly to the ground. However, aerosolized particles smaller than 5 μm contaminate the air and can levitate indoors where the air is not exchanged and disinfected for several hours. In addition to PPE, aerosol extractors and other elements preventing significant air contamination in the clinic, it is therefore appropriate to include other forms of air conditioning, in our case hooded UVC emitters combined with diffusers of biocidal oils, suitable for combination with UVC sterilization.

#### 2.4.4. Air-Processing Elements (UVC + Virucidal Diffusers)

Sterilization of the entire air volume of one separate room in clinic with volume of 13 m^3^ will take approximately 25~30 min (time intervals between patients). SARS-CoV-2 virus particles are rapidly inactivated by UVC radiation. Two hardware key elements of the presented prospective biosafety setting are UVC and Diffuser.

(A) Germicidal radiator PROLUX G30W A/SPH01:
  - UVC lamp life of 8000 h;
  - For two-shift operation endurance 550 days (2 × 7.5 h);
  - 1 emitter cycle is sufficient to sterilize a volume of 5.5 m × 4 m × 3.0 m;
(B) Aroma Pro Mini—professional diffuser (aroma atomizer).
  - For air conditioning;
  - Capacity up to 1000 m^3^ (www.NewAroma.sk accessed on 15 December 2021);
  - Possibility to choose from several certified disinfectant oils;
  - Disinfectant is present in the mixture in 3 weight percent at an emission of 5 mL per hour (adjustable) to form an invisible aerosol dispersion in the air.

### 2.5. Protocol Development and Evaluation

The protocol has been in development from 8 February 2020 until 1 June 2020 (5 months). The unchanged protocol in place was from 1 June 2020 until 30 November (18 months). The protocol evaluation with auto-locked online smart forms was performed between 1 December and 12 December.

### 2.6. Protocol strengths and Weaknesses

Strengths:

- Low cost, affordable, effective, sustainable
- Does not require specific rebuilding of the current dental set-ups
- Dimensioned for highly transmissible strains coming after Omicron
- Not dependable on vaccination status or unreliable testing results

Weaknesses:

- Difficult to evaluate clinical reliability:
  ○ The only possible way is feedback form (bias);
  ○ Larger sample needed;
  ○ Other studies for comparison are needed;
- Requiring extra time gaps between patients in the same room;
- Possible biosafety overkill;
- Unknown performance under unexplored highly transmissible variants.

## 3. Results

### 3.1. Descriptive Results

From 160 relevant patients, the online smart form was filled out and submitted 115 times. There were eight responses in English the rest were in Slovak. The special fingerprint feature identified three duplicate answers that were removed. The result was 111 valid responses (69.37%). All answers are in the table that is available online in the public repository.

**Figure 4:**
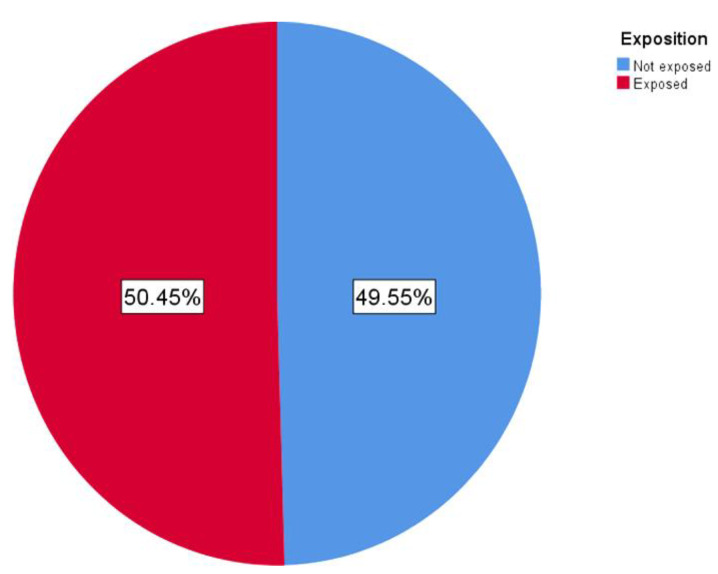
This graph represents distribution of exposed vs. non exposed patients to someone diagnosed with COVID-19 during the last 18 months.

**Figure 5:**
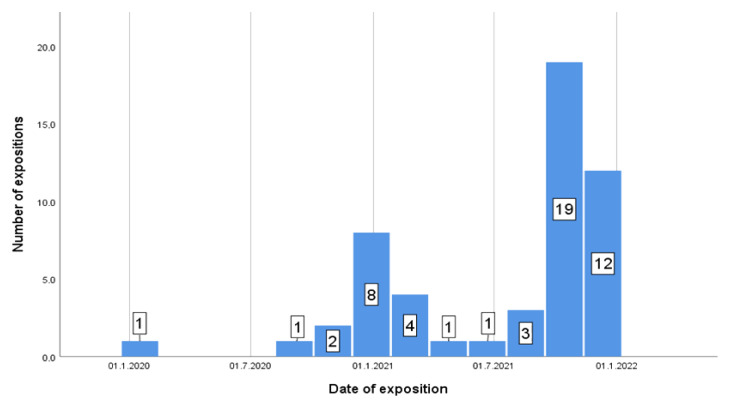
Frequency of exposure to an infected person by the date.

Approximately half of the responding patients were exposed to an infected person with COVID-19 (Figure 4). From 111 patients, exactly 56 (50 5%) were directly exposed to SARS-Co-2 infection and 55 (49 5%) were not (during the last 18 months). Dates of these exposures are shown in Figure 5.

**Figure 6:**
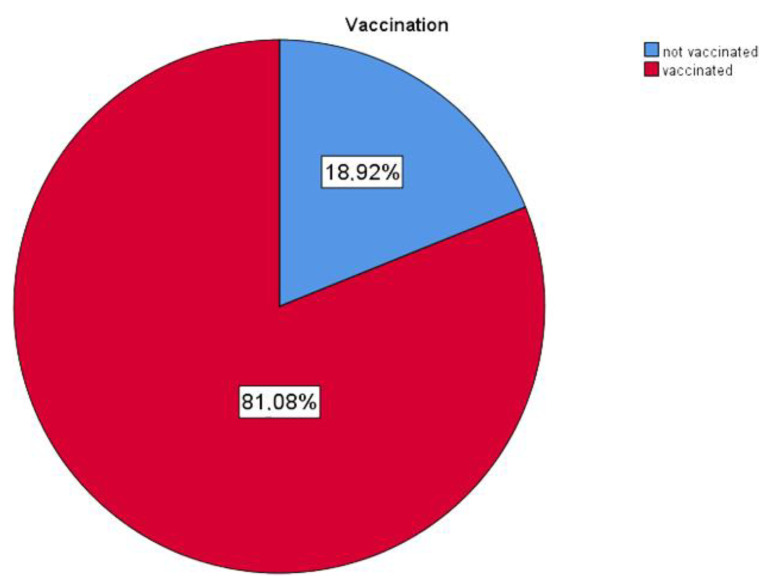
Vaccination distribution in patients.

Of the responding patients, 90 (81%) were vaccinated (Figure 6 and Table 1); however, only 14 had had a Pfizer booster (3rd) shot.

Fifty-eight were vaccinated with two shots of Pfizer, seven with Moderna, four with Janssen and eight with AstraZeneca.

Forty-five (50%) of all vaccinated patients received their last shot until June 2021.

Nine patients reported the possibility of being infectious during their visit at the clinic.

Thirty-eight responders got infected with the SARS-CoV.2 virus.

Thirty- seven responders rejected the possibility that they got infected at the clinic under the evaluated protocol. Only one of them considered this as the possibility with the lowest offered level of confidence.

**Figure 7:**
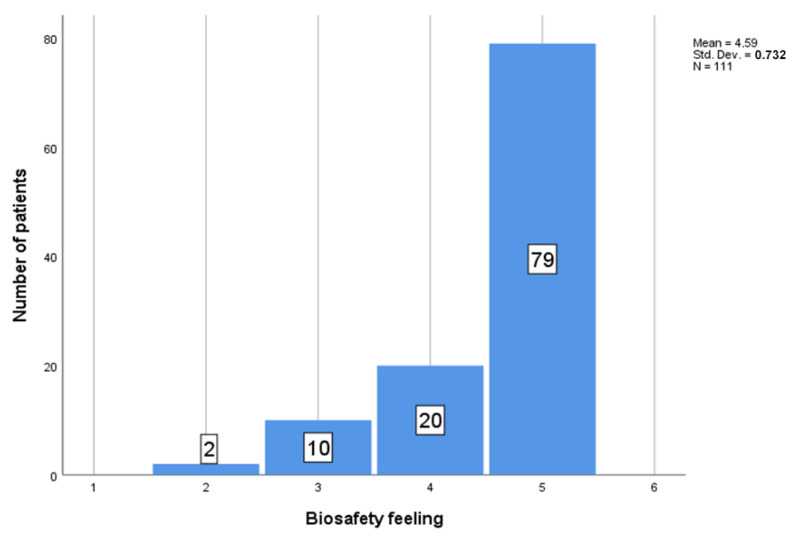
Biosafety feeling records by value.

Regarding the question about the feeling of biosafety experienced by the patients during the dental procedure (Figure 7), the patients’ feelings were evaluated on a scale from 1 to 5, where the 1 represents “no safety feeling” to 5 “very safe”. From 111 patients, the mean feeling was M = 4.59, SD = 0.732.

**Figure 8:**
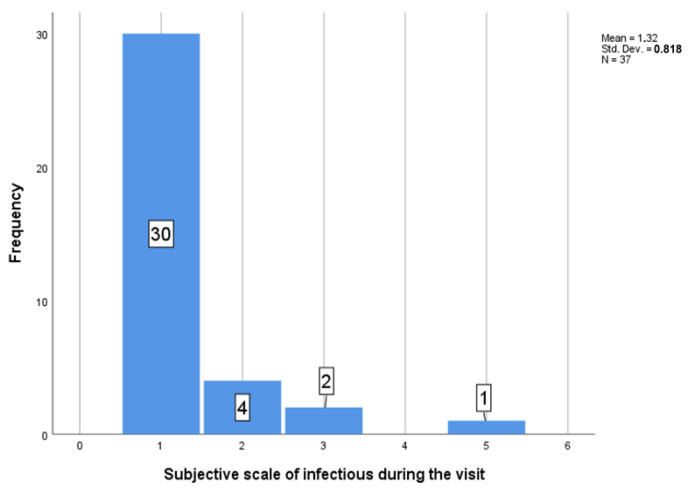
Number of subjective infectious status during visit.

**Figure 9:**
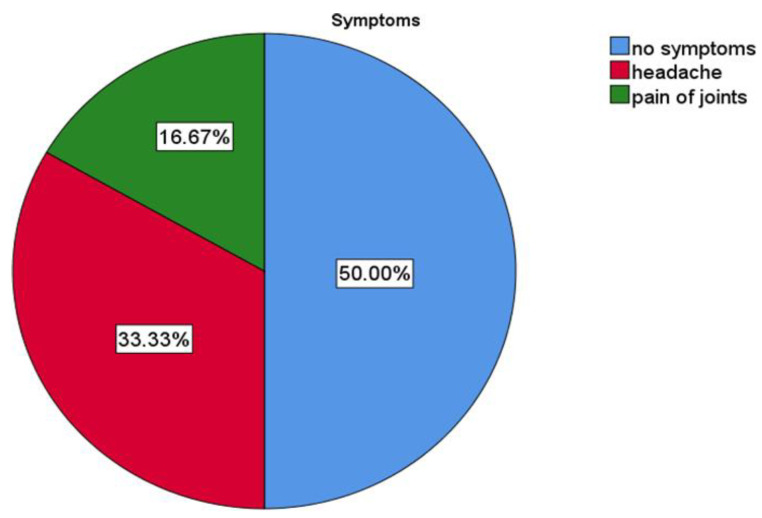
Potential infectious patients during the visit and spectrum of their symptoms.

Thirty-seven patients answered the question “Could you have ever visited our clinic already infected?” (Figure 8). The scale available was from 1 (*no, there was no chance of being infectious during the visit*) to 5 (*yes, I was infectious during the visit*). M = 1.32, SD = 0.818. All the infectious patients visiting the clinic were asymptomatic, as otherwise they would not have got through the initial entry filter (Figure 9).

### 3.2. Graphical Interpretation of Clinical Evaluation of the Protocol

Out of 111 patients, 56 (50.5%) were exposed to SARS-Co-2 infection and 55 (49.5%) were not (Figure 4). The incidence of exposure to an infected person is shown in Figure 5.

The one-sample t test revealed a significant difference in the distribution of the exposures *t* (51) = 7,103,185, *p* < 0.001 during the time (M = 14.7.2021; SD = 162 d).

The independent sample t test showed the significantly higher number of exposures in the unvaccinated group (N = 11, M = 3.91, SD = 1.3) compared to in the vaccinated group (N = 45; M = 2.42, SD = 1.438) *t* (54) = 3.127, *p* = 0.003.

There was no significant correlation and a low Pearson‘s correlation (almost significant) between the date of exposition and number of the protentional contacts with SARS-Co-2 r = 0.265, N = 52, *p* = 0.058.

### 3.3. Biosafety Protocol

The safety feeling of the patients was recorded on a scale from 1 to 5, where the 1 represents “no safety feeling” to 5 “very safe”; from 111 patients, the mean feeling was M = 4.59, SD = 0.732 (see Figure 7 and Table 2).

Between the two groups of unvaccinated patients—one of which was named “Never got the vaccine against SARS-Co-2” (N = 5, M = 5, SD = 0) and the other was “Unvaccinated, but still waiting” (N = 16, M = 4.5, SD = 0.730)—there was no significant difference between the biosafety feeling *t* (19) = −1.504, *p* = 0.149.

There was no statistical difference in biosafety feeling between the group of vaccinated patients and the patients who had not had a booster dose (N = 78, M = 4.56, SD = 0.783) and those who had had a booster dose (N = 12, M = 4.67, SD = 0.492).

Thirty-seven patients answered the question “Could you have ever visited our clinic as already infected?”. There was scale, which divided the risk of infectious status to 1 (no, there were no chance to be infectious during the visit), to 5 (yes, I was infectious during the visit) M = 1.32, SD = 0.818.

Of the patients who answered the question of possible infectious status during the visit (N = 6) (scale of potential risk was higher than 1), most recorded that they had no symptoms during the visit N = 3 (50%), but others noted that they had headache N = 2 (33.3%) and pain of joints N = 1 (16.67%) (Figure 8 and Figure 9).

There was medium correlation between number of exposures and potentially infectious patients r (28) = 0.402; *p* = 0.034.

## 4. Discussion

The evaluated prospective setting for orthodontic workflow is based on a combination of well-researched technologies and known virucidal effects. Together, this combination is simple and sustainable. Despite the results, which preliminarily suggest its virucidal effectivity of such a prospective setting, these shall be considered as preliminary data for a larger study.

Evaluated subjective feedback from pseudonymous questionnaires showed that, under this prospective setting, infected patients were treated with high probability. Additionally, data suggest that chances of SARS-CoV-2 cross infection occurrence in the monitored clinical environment were extremely low.

With an awareness of the limitations of single-arm, single-center studies, it shall be emphasized that practice guidelines should rarely, if ever, be based on evidence from single-center trials and this study shall encourage clinicians from the dental community to engage in further and wider evaluation of such prospective settings. The combination of UVC and dispersed oil in virucidal air-processing might evolve in the very near future to a sustainable model for biosafe dental care. Dispersion of aerosols during dental therapy and other procedures and technologies to reduce the contagion among dentists have been well researched [7,110,111]. However, there is currently no comparative data available about how frequently patients become infected during orthodontic appointments or other biosafety efficiency.

Further interpretation of the study results shows that from 115 online form responses, one was probably intentionally invalid as it repeatedly referred to triple vaccination even before third shots were available in the EU, as well as references to events out of the observed 18-month period and it entered various other oxymorons. This response has been evaluated as invalid.

Approximately every second responder had a history of recent COVID-19 exposition. Dates of these exposures (Figure 5) correlate with the Slovak regional pandemic situation and it is clear to see the difference between the previous and the current wave.

Forty-five (50%) of all vaccinated responders received their last shot before June 2021, so with over 6 months since last vaccination, their immunity from vaccines against the Omicron variant will not be probably relevant.

The evaluation of the protocol reveals nine patients reporting the possibility of them being infectious during their visit to the clinic. There was no incident of personnel becoming infected by patients or within work. Only two of the personnel were infected with COVID-19 in the past. Both cases were from well-known family sources.

Of the 38 infected responders, 37 rejected the possibility that they were infected at the clinic in the monitored period. Only one of them considered this as a possibility, but with the lowest offered level of confidence. There were five levels of confidence in this parameter:

1. Certainly not! 0%;
2. 25%;
3. Maybe, 50%;
4. 75%;
5. I am sure I got it there 100%.

Thirty-seven patients answered the question “Could you have ever visited our clinic already infected?” (Figure 8). All the infectious patients visiting the clinic were asymptomatic, as they would not have got through the initial entry filter (Figure 9).

In this paper, the authors have presented a biosafety protocol with further context to orthodontic care facing the Omicron variant with new epidemiological properties. With this new variant, a higher transmissibility and lower protection from vaccines can be anticipated, albeit with possibly milder clinical symptoms [79,81,82,83,84,85,87,89,90]. So, there will be more likelihood of having an asymptomatic carrier in a dental procedure in the near future than there is today.

The results of the survey have demonstrated that patient entry-symptomatic-screening prevented symptomatic patients present in the dental procedures. Survey also revealed that most of the vaccinated patients have probably very low or nearly no protection from vaccines [45,77,79,81,112].

The weakness of this study is the clinical evaluation of the clinical performance of the true safety of this biosafety protocol. The online survey does not show a representative sample; it depends on the self-assessment of the responders. Additionally, there is a high probability that responders are the more responsible part of the targeted group.

The results can be interpreted from the perspective of previous studies presented in the Introduction chapter as supporting the validity of the protocol. While they do not prove it to be safe, they do prove that it is not unsafe. Wider and multicentric research is necessary to prove the reliability of these settings. Ideally, research should be carried out with similar clinics not following the protocol, but working in the similar geographical location and intensity.

Approximately half of the responding patients were exposed to person infected with COVID-19; however, this is self-assessment evaluation. It is interesting that the dates of these exposures correlate with the Slovak regional pandemic situation in those times. It is also an obvious difference between the previous and the current wave. The frequency of exposures during the current wave is higher than in the previous one, when more strict lockdowns were implied. Fifty percent of all vaccinated patients had received their last shot before June 2021, so with over 6 months since the last vaccination, their immunity from vaccines against the Omicron variant will not be probably relevant [45,81].

The more transmissible Omicron variant defines a new chapter of the COVID-19 pandemic. As it is spreading at a rate unseen with any of the previous variants, there is a concern that people are dismissing Omicron as mild, not learning from the recent past. Even if Omicron does cause less severe disease, the sheer number of cases could once again overwhelm unprepared health systems [113].

Omicron contains mutations associated with higher levels of immune escape, higher transmissibility, and an improved ability to bind cells. However, there are also many mutations within the new variant that are not yet understood. As the experts have no idea what these new mutations do yet, it is logical to stay cautious and responsible [113].

Predictions of the practicality and efficiency of this prospective setting for dental care are difficult, as they face only the preliminary findings about the Omicron variant, and society’s attitudes are changing. Experts are currently facing considerations of Omicron as a possible pandemic-ender, with some people including some politicians willing to take the risk for the sake of the economy. Some people are even willing to voluntarily be infected, despite the Omicron variant’s known risks of infection, such as impact on the nervous system, heart tissue or the risk of long COVID-19 are known. It remains unclear whether Omicron will have any of the “silent” effects seen with earlier variants, such as self-attacking antibodies, sperm impairments and changes in insulin-producing cells.

Only time will reveal if there are other hidden risks for our health. In this regard, only recently have researchers found strong evidence that it is an infection with the Epstein–Barr virus—a particularly ubiquitous member of the herpesvirus family, best known for causing mononucleosis and triggering multiple sclerosis (MS). Infection with Epstein–Barr increased the likelihood of developing multiple sclerosis, by more than 32-fold [114].

However, as this new era puts current vaccines into a different perspective, masks, ventilation, and hygiene remain unaffected. Vaccines are tools that have the greatest impact when they are used to protect those who are most at risk. They are the last line of our defense. Today, vaccines cannot be considered as a substitute for masks, distancing, ventilation, or hand hygiene. It also seems logical that Omicron-like strains are here to stay. It can be fought with measures that work today and that must be sustainable. This presented biosafety protocol addresses higher risks suggested by preliminary observations that indicate that Omicron spreads faster and escapes antibodies more readily than previous variants. Loss of smell and taste is clinically frequent in older variants; now, with Omicron, sore throat and night sweats are reported frequently. The protocol anticipates that an increase in reinfections and cases of mild breakthrough infections in people who are vaccinated is highly probable [89].

## 5. Conclusions

A clinical evaluation of the introduced prospective biosafety settings for dental care and education suggests a possible sustainable solution for the next pandemic season.

The results of this work indicate the perspective application of combined procedures and technologies focused particularly on virucidal air-processing with UVC and oil dispersion as well as other technologies such as AI and telemedicine in confrontation with air-borne SARS-CoV-2 variants.

The presented prospective setting in prevention of COVID-19 was evaluated using 111 responses, suggesting that nine patients were treated as infectious asymptomatic carriers (with high probability), but no cross infection has been identified.

Despite the results suggesting the virucidal effectivity of this sustainable biosafety prospective setting, these shall be considered only as preliminary data for a larger study. Recognizing the limitations of single-arm, single-center studies, it shall be highlighted that practice guidelines should rarely, if ever, be based on evidence from single-center trials and this study shall encourage clinicians from the dental community to engage in further and wider evaluation of described technologies and procedures.

While more infectious Omicron-like strains do appear to be clinically less severe compared to Delta, their long-term effects are still unknown and dental professionals must not risk patients’ infection [115].

## Figures and Tables

**Table 1:** Vaccination status of all responses.

Status	Frequency	Percent
not vaccinated	21	18 9
vaccinated	90	81 1
Total	111	100 0

**Table 2:** Biosafety feeling of patients during dental procedures, full data available in supplementary materials.

					95% Confidence Interval for Mean			
	N	Mean	Std. Deviation	Std. Error	Lower Bound	Upper Bound	Minimum	Maximum
disease free	74	4.55	0.743	0.086	4.38	4.73	2	5
Ab +	3	4.67	0.577	0.333	3.23	6.10	4	5
PCR/Ag +	30	4.73	0.583	0.106	4.52	4.95	3	5
multiple	3	3.67	1.528	0.882	−0.13	7.46	2	5
other	1	5.00	0.0	0.0	0.0	0.0	5	5
Total	111	4.59	0.732	0.069	4.45	4.72	2	5

## Data Availability

We fully adhere to Data Availability Statements in section “MDPI Research Data Policies”.

## Supplementary Materials

The following are available online at https://docs.google.com/spreadsheets/d/1lznYWB32gTb282v4HJbQRY_Uw42xXnBhNj-vnmvJvKE/edit?usp=sharing (accessed on 18 May 2022).
